# Emerging Predictors of Obstructed Labour in a Single Nigerian Centre Population of a Low Resource Setting

**Published:** 2022-10-01

**Authors:** Charlotte B. Oguejiofor, Chinedu J. Ezugwu, George U. Eleje, Ekene A. Emeka, Josephat C. Akabuike, Joseph C. Umeobika, Onyecherelam M. Ogelle, Osita S. Umeononihu, Ahizechukwu C. Eke

**Affiliations:** 1 Department of Obstetrics and Gynecology, Faculty of Medicine, Nnamdi Azikiwe University, Awka, Nigeria; 2 Department of Obstetrics and Gynecology, Nnamdi Azikiwe University Teaching Hospital, P.M.B. 5025, Nnewi, Nigeria; 3 Department of Family Medicine, Faculty of Medicine, Nnamdi Azikiwe University, Awka, Nigeria; 4 Department of Obstetrics and Gynecology, Chukwuemeka Odumegwu Ojukwu University Teaching Hospital, Awka, Nigeria; 5 Division of Maternal-Fetal Medicine, Department of Gynecology and Obstetrics, The John Hopkins University School of Medicine, Baltimore, Maryland, United States

**Keywords:** Duration of labour, low income, predictors, teenage pregnancy, unbooked, nulliparity, uterine rupture

## Abstract

**Background and Objective:**

Despite the stigma attached to obstructed labour in Nigeria, it has remained largely uninvestigated. Study determined the prevalence, emerging predictors, management modalities and complications of obstructed labour, compare them with cases without obstructed labour who delivered within the same period.

**Materials and Methods:**

A retrospective study and case-controlled analysis of obstructed labour managed at Nnamdi Azikiwe University Teaching Hospital, Nnewi, South-East, Nigeria were undertaken. One control per case was randomly selected from the remaining births by selecting the non-obstructed labour cases. Bivariate analysis was performed by the Chi-squared test and conditional logistic regression analysis was used to determine variables associated with obstructed labour. Statistical significance was accepted when the p<0.05.

**Results:**

Of all the 5,301 deliveries during the study period, 80 cases of obstructed labour were recorded, giving a prevalence of 1.5%. Only 73 case files were available with complete information for the study’s further analysis. A conditional logistic regression analysis, the risk factors were teenage pregnancy (p<0.001, Adjusted Odds Ratio (AOR):5.43, 95% Confidence Interval (CI):1.20–8.05), unbooked status (p<0.001, AOR:0.01, 95%CI:0.00–0.02), nulliparity (p<0.001, AOR:4.15, 95%CI:2.42–7.25), short stature (p<0.001, AOR:44.74, 95%CI:19.51–113.53) and birth weight (p<0.001, AOR:4.52, 95%CI:2.69–7.71). The case fatality rate was 5.5% and the perinatal mortality rate was 21.9%.

**Conclusion:**

Majority obstructed labour have high maternal morbidity and perinatal mortality.

## INTRODUCTION

Labour is obstructed when there is no further progress in the labour progress of cervical dilatation and descent of the presenting part despite adequate uterine contraction due to mechanical problems^1,2^. Obstructed labour is an absolute and not a relative obstetric phenomenon and it refers to neglected feto-pelvic disproportion in labour. It is a life-threatening obstetric complication associated with significant materno-fetal morbidity and mortality^3–5^. It accounts for approximately 8% of all maternal deaths in developing countries like India^4^. Maternal mortality from obstructed labour is largely the result of a ruptured uterus or puerperal infection, whereas perinatal mortality is mainly due to asphyxia^1,3,5–11^.

The prevalence of obstructed labour varies worldwide, even within the same country. It is commoner in low-income countries where poverty, malnutrition and early marriage are very common^1^. It is virtually unknown in high-income countries in this present day obstetrics. In India, the incidence of obstructed labour is 1.6%^5^. The incidence of obstructed labour in Ethiopia is 12.2%^3^ while in Uganda it is 10.5%^2^. In Nigeria, the prevalence ranges from 0.78–4%^1,8,9,11^.

Obstructed labour is commoner in those with short stature (<1.52 m), unbooked with minimal or antenatal care, low socio-economic class and abnormal gait from previous pelvic fracture^5,10^. The causes of obstructed labour include fetopelvic disproportion, mal-presentation, pelvic tumours and abnormalities in the uterine cavity as in the septate uterus^1,2,12–14^.

Maternal complications of prolonged obstructed labour include sepsis, metabolic and electrolyte derangement, exhaustion, ruptured uterus, primary postpartum haemorrhage, obstetrics fistulas, osteitis pubis, gait abnormalities due to nerve injuries, secondary amenorrhea, secondary infertility, economic, psychological and social impairment^1–3,5,8^. Fetal complications are the sequel to chorioamnionitis, uterine hyperstimulation and uterine rupture and unskilled attempts at vaginal delivery. These include fetal distress, birth asphyxia, neonatal sepsis, intrauterine fetal death and neonatal death^1,2,5,13,15–18^.

Despite the stigma attached to obstructed labour in Nigeria, it has remained largely uninvestigated. Against this framework, the present study was conducted to determine the prevalence, clinical presentation, emerging risk factors and pregnancy outcomes of obstructed labour cases in Nnamdi Azikiwe University Teaching Hospital, Nnewi, Nigeria and compare them with cases without obstructed labour who delivered within the same period in the same hospital. This will help illuminate the changing trend in the risk factors and pregnancy outcomes in cases of obstructed labour as well as justify the policy of questioning the rigid limits currently applied in clinical practice for the assessment of prolonged first or second stages that warrant obstetric interventions^19^.

## MATERIALS AND METHODS

### Study area:

This was a retrospective study and case-controlled analysis of obstructed labour at NAUTH Nnewi, South-East, Nigeria from 1st January, 2013 to 31st December, 2017. The hospital serves as a major referral Centre in South-East Nigeria. The names and case files of all the cases of obstructed labour were identified from the records in the case notes, operating theatre and the labour ward registers. The labour ward register also provided information on the total number of deliveries within this study period.

### Research protocol:

Their case files were retrieved from the Medical Record Department of the Hospital. The information on age, parity and marital status, the highest level of education, maternal height and occupation were extracted from the case files in a non-electronic format. The other information extracted included duration of labour (first and second stages), type of surgery performed (whether a caesarean section or laparotomy) and maternal as well as fetal complications. Any woman who has attended and registered at least one antenatal clinic in the study hospital before delivery or labour is said to be booked. For case or study groups, included only women with the diagnosis of obstructed labour and managed in the obstetrics and gynaecology unit of the hospital. Cases of antepartum haemorrhage were excluded. One control per case was randomly selected from the remaining births by selecting the non-obstructed labour case admitted to the labour suit within the same 24 hrs period in active labour.

### Ethical clearance:

Ethical clearance was obtained from the Teaching Hospital Ethics Committee.

### Procedure:

A thorough scrutiny of the delivery records of the obstetrics unit as well as the records of the medical records department and the special baby unit of the hospital was done. This was achieved by checking their names, case file numbers, computer numbers and diagnosis at the presentation at the labour ward as well as the subsequent management of their babies if admitted to a Special Care Baby Unit (SCBU). The pro-forma was initially used for data collection which was transferred to a data sheet before transferring to SPSS software. Social class stratification was determined^20^: Classes 1, 2 and 3 were considered upper class, middle class and lower social class, respectively. Tertiary education was defined as polytechnic or university education. The total deliveries as well as live births and maternal deaths during the study period were determined. The perinatal outcomes were also determined by identifying the APGAR score at delivery, birth weight and the conditions of the babies on or before 7 days of life at an SCBU or condition at discharge. If more than one outcome was found for the cases of multiple pregnancies, the worst outcome was used for analysis. The diagnosis of obstructed labour was made clinically. In this study, a case was defined to be obstructed using the following criteria: a cervical dilatation ≥6 cm with ruptured membranes, having adequate contractions lasting more than 4 hours with no change in cervical dilatation in the first stage of labour or for the second stage of labour, an arrest was defined as a delay of greater than 2 hours with adequate uterine contractions, with at least any two of the following obvious signs of severe obstruction: Caput formation, subconjunctival haemorrhage, oedematous vulva and Bandl’s ring^21^.

A laparotomy was said to be performed when a surgical procedure involved a large incision through the anterior abdominal wall to gain access to the abdominal cavity. Birth asphyxia was defined as one minute APGAR score of less than 6. Puerperal sepsis was diagnosed based on this criteria: Infection of the genital tract occurring at any time between the obstructed labour and the 42 day postpartum in which at least two of the following were present: Pelvic pain, fever (i.e., oral temperature ≥38.5°C/101.3°F on any occasion), abnormal vaginal discharge and sub involution of the uterus (delay in the rate of reduction of the size of the uterus of <2 cm per day within the first 8 days).

In all cases, sepsis was prevented by the administration of antibiotics (ampicillin/sulbactam 1.5 g and metronidazole 500 mg given intravenously for 72 hrs, then orally for 5 days). The urethral Foley catheter was left *in situ* for 14 days in all cases.

### Statistical analysis:

Descriptive statistics were used in calculating percentages, means and standard deviation. Data was entered after checking completeness, cleaning and coding into computer SPSS version 23. Data analysis was done and the results were displayed in tables. To determine the relationship between obstructed labour and associated risk factors, the Chi-squared test, Fisher’s exact test and t-test whenever appropriate, were performed in the bivariate analysis. Conditional logistic regression was employed in the multivariate analysis to determine variables associated with obstructed labour, while controlling for other confounding variables (age, booking status, parity, mode of delivery, socio-economic class and gestational age at delivery). In this analysis, the Odds Ratio (OR) and confidence interval was set at 95% (95% CI) and p<0.05 was considered significant.

## RESULTS

In this 5 years review period, there were a total of 5,301 deliveries. Out of this, 80 patients had obstructed labour. This gives a prevalence of 1.5%. However, only 73 case files were available with complete information for the study, giving a retrieval rate of 91.3%.

Of all the 73 study cases, only 1 (1.4%) laboured in the study hospital, while 74 (98.6%) laboured at home or the referral hospital before the presentation. The average duration of labour (first and second stages) in the obstructed labour group was 27.7±6.2 hrs while the duration of labour (first and second stages) for the non-obstructed labour group was 14.1±3.5 hrs (p<0.05). The association between obstructed labour and women’s characteristics based on a bivariate test is shown in Table 1. In the obstructed labour versus non-obstructed labour groups, 21(28.8%) vs 5 (6.8%), 9 (12.3%) vs 70 (95.9%) and 19 (28.4%) vs 33 (45.2%) were age less than 20 years, booked and has high social class, respectively. The statistically significant differences were observed for teenage age (less than 20 years), unbooked status, parity, socio-economic status and birth weight (p<0.05, for all).

Regarding obstructed labour, the obstructed group and the non-obstructed group were compared using multivariate logistic regression, while controlling the effects of possible confounding variables, such as age, booking status, parity, socio-economic class, maternal height and birth weight. Table 2 shows the association between obstructed labour and women’s sociodemographic and obstetric characteristics based on bivariate tests and multivariate logistic regression. In the obstructed labour versus non-obstructed labour groups, 21(28.8%) vs 5 (6.8%), 9 (12.3%) vs 70 (95.9%) and 19 (28.4%) vs 33 (45.2%) were age less than 20 years, booked and has high social class, respectively. The results showed that the risk factors of obstructed labour were teenage pregnancy (p<0.001, adjusted odds ratio (AOR): 5.43, 95% confidence interval (CI):1.20–17.13), unbooked status (p<0.001, AOR:0.01, 95% CI:0.00–0.02), nulliparity (p<0.001, AOR:4.15, 95% CI:2.42–7.25) and birth weight (p<0.001, AOR:4.52, 95% CI:2.69–7.71).

Table 3 shows the causes of obstructed labour observed in women. Cephalopelvic disproportion was responsible for the majority, 53 (72.6%) of cases.

Table 4 shows the type of surgery performed among women with obstructed labour. Caesarean section 61 (83.6%) was the commonest surgery performed. Table 5 shows the maternal outcome and complications of obstructed labour. Abdominal distension 15 (20.5%), uterine rupture 11 (15.1%) and postpartum haemorrhage, 9 (12.3%) were the three most common complications. There were four maternal deaths, giving a case fatality rate of 5.5%.

Table 6 shows the fetal outcomes and complications of obstructed labour. The commonest neonatal complication of obstructed labour was birth asphyxia 23(31.5%). The stillbirth rate was 14/73 (19.2%), the perinatal mortality rate (stillbirth (n = 14) and early neonatal death (n = 2)) was 16/73 (21.9%), while the live birth rate (total births (n = 73) minus stillbirth (n = 14)) was 59/73 (80.8%).

## DISCUSSION

The principal findings in this study were that the prevalence of obstructed labour was 1.5% and the emerging risk factors were teenage pregnancy, unbooked status, nulliparity and high birth weight. Complications were observed in 79.5% of the women and the common morbidities were abdominal distension, uterine rupture, postpartum haemorrhage, wound sepsis and puerperal sepsis. The case fatality rate was 5.5% and perinatal mortality was 21.9%.

As revealed in this study, the prevalence of obstructed labour was 1.5% which is comparable to the Indian study which gave a prevalence of 1.6%^6^. This prevalence was however lower than some other previous Nigerian studies^1,3,11^. However, it is higher than 0.78% reported by Jeremiah and Nwagwu^9^ in Port Harcourt, Nigeria. This decreasing trend in prevalence is probably a reflection of improvement in antenatal and intrapartum care.

In this study, at conditional logistic regression analysis, the emerging predictors of obstructed labour were teenage pregnancy, unbooked status, nulliparity, maternal height and birth weight (p<0.001). This finding is similar to that reported by Bako *et al*.^1^ in Maiduguri, Nigeria and Jeremaiah and Nwagwu^9^ in Port Harcourt, Nigeria, but differs from Musaba *et al*.^21^ studies in Uganda. In Musaba *et al*.^21^ studies, unbooked status and birth weight were not predictors of obstructed labour. Nevertheless, health education is suggested for all women but especially the primigravida who have untested pelvis before embarking on the labour process. As observed in this study, the majority avoided the hospital environment for antenatal (87.7%) and intrapartum care (98.6), preferred to deliver at the maternity home and also by the traditional birth attendants, but only to be referred to the study hospital.

Cephalopelvic disproportion was the major cause of obstructed labour (72.6%) which is comparable to the study done in Maiduguri, Nigeria by Bako *et al*.^1^ (65.37%) but higher than the reports from India by Mondal *et al*.^5^ (55.6%), Islam *et al*.^12^ (47.5%) from Bangladesh. It is also similar to the same study done in Sokoto, Nigeria, by El Nwobodo and Ahmed^11^ which revealed cephalopelvic disproportion as a major culprit for obstructed labour in 78.5% of cases.

In obstructed labour, there is no place for a wait-and-watch policy. In this study, the majority of cases (83.6%) were delivered by emergency lower segment caesarean section while laparotomy plus repair of the uterus and laparotomy plus subtotal hysterectomy accounted for 13.7 and 2.7% of the surgeries performed, respectively. These were performed for cases of uterine rupture. This finding is akin to Bako *et al*.^1^ (80.97%) in Maiduguri, Nigeria, Mondal *et al*.^5^ (85.9%) in India and Ngongo *et al*.^14^ (81.8%) in three tertiary hospitals in South-East Nigeria. This is however higher than that reported by Islam *et al*.^12^ (78.1%) from Bangladesh. Unlike the reports by Sikka *et al*.^15^ from India and Umar *et al*.^16^ from Sokoto, Nigeria which reported 0.26 and 0.31% of destructive deliveries respectively, this review did not record any case of destructive delivery among those with confirmed intrauterine fetal death. This could be due to current practice.

Although obstructed labour is completely a preventable entity, in this study, the maternal case fatality rate was 5.5%. Both uterine rupture and wound sepsis were responsible for the mortalities. This finding is corroborated by reports of Jeremiah and Nwagwu^9^ (7.2%) and Nwobodo and Ahmed^11^ (8.3%). Similar to this study, Bako *et al*.^1^ have also revealed that uterine rupture was the most common cause of maternal mortality in obstructed labour.

In terms of complications of obstructed labour, abdominal distension (19.2%), uterine rupture (15.1%) and postpartum haemorrhage (12.3%) were the most common complications. This finding is different from the report by Bako *et al*.^1^ in Maiduguri, Nigeria, Jeremiah and Nwagwu^9^ in Port Harcourt, Nigeria and Nwobodo and Ahmed^11^ in Sokoto, Nigeria, which showed that the most common complication was wound sepsis. In another study by Mondal *et al*.^5^, the commonest maternal complications included pyrexia (49.8%), postpartum haemorrhage (33.9%), urinary tract infection (10.9%), sub involution (9.3%) and wound infection (7.7%).

Perinatal mortality in various studies is as follows Wonde and Mihretie^17^ (36.2%), Islam *et al*.^12^ (24.76%), Nwobodo and Ahmed^11^ (55.4%) and Mondal *et al*.^5^ (22.7%). In the present study, the perinatal mortality rate was 21.9%, the stillbirth rate was 19.2% and the live birth rate was 80.8%. These findings were similar to that reported by Mondal *et al*.^5^ which showed that the still birth rate was 18.21% and the live birth rate was 81.8%.

The most common neonatal complication of obstructed labour in this study was birth asphyxia (31.5%). This is similar to the report by Mondal *et al*.^5^ in India that birth asphyxia was the commonest neonatal complication of obstructed labour (29.7%). From the present study, other neonatal complications of obstructed labour included neonatal jaundice and neonatal sepsis.

As revealed in this study, 98.6% of women that had obstructed labour laboured at home or the peripheral hospital before the presentation. This finding is alarming especially in this era of modern technology. This no doubt has revealed the extent of the resulting poor outcomes in the women that had obstructed labour accounted for by the care they received or failed to receive when they were outside of the study hospital.

Additionally, the findings of this study do have an important lesson for practitioners who work in high-income settings. Although there is considerable controversy about the appropriate length of labour that should be allowed, labour that is much longer than has generally been considered reasonable and safe is now becoming advocated by new guidelines, especially in the United States^19^. Since the average duration of the labour in the obstructed labour group was more than 26 hrs, current findings can give pause to anyone advocating excessively long labour, regardless of whether they might be considered to be obstructed^19^. Therefore, recognizing abnormal labour progression and commencing suitable intervention is vital because prolonged labour is associated with amplified risks for operative delivery and maternal and neonatal morbidity.

The strengths of this study were the higher number of investigated variables, application of standardized protocols for all cases, homogeneity of study groups and first-time comparison of outcomes with normal cases for an obstetric complication. Secondly, all the women included as cases had well-defined criteria for diagnosis: When there was documented failure of descent of the fetus in the birth canal for mechanical reasons despite good uterine contractions when the women. On the other hand, the limitations of this study were a relatively small number of cases, retrospective design and single-centre experience. This study could not determine the exact duration of the first stage (latent phase and active phase) and second stage of labour in the obstructed labour group because almost all of the women laboured outside the study hospital, could only determine the overall combined duration of the first and second stages of labour as was assessed via the clinical history given by the women when they presented. The prospective study design will allow clarification of unclear documentation and thereby improve data quality.

## CONCLUSION

The majority of cases of obstructed labour have high perinatal mortality and maternal morbidity. Teenage age, unbooked status, nulliparity, short stature and high birth weight were emerging significant predictors, while all women had operative deliveries and a significant number of feto-maternal morbidities. Since women in the obstructed labour group had a significantly longer duration of labour, this study calls for a review or pause to the policy of advocating excessively long labour in low-income countries, regardless of whether they might be considered to be obstructed.

## Figures and Tables

**Table 1:** Association between obstructed labour and women’s characteristics based on bivariate test

Variables/subgroup	Obstructed group (n = 73)	Non obstructed group (n = 73)	p-value
**Age <20 years**			
Yes	21 (28.8)	5 (6.8)	*<0.001
No	52 (71.2)	68 (93.2)	
**Booking status**			
Booked	9 (12.3)	70 (95.9)	*<0.001
Unbooked	64 (87.7)	3 (4.1)	
**Parity**			
Nulliparous	38 (52.1)	15 (20.5)	*<0.001
Parous	35 (47.9)	58 (79.5)	
**Socio-economic class** *			
High	19 (28.4)	33 (45.2)	*0.039
Low	48 (71.6)	40 (54.8)	
**Maternal unemployment**			
Yes	63 (70.3)	54 (77.1)	0.062
No	10 (29.7)	19 (22.90)	
**Maternal height (meters)**			
≤1.52	53 (72.6)	4 (5.5)	*<0.001
>1.52	20 (27.4)	69 (94.5)	
**Gestational age at delivery**			
≥40 weeks	40 (54.8)	35 (47.9)	0.408
<40 weeks	33 (45.2)	38 (52.1)	
**Birth weight**			
≥4.0 kg	45 (61.6)	19 (26.0)	*<0.001
<4.0 kg	28 (38.4)	54 (74.0)	

* Only 67 were married

**Table 2:** Association between obstructed labour and women’s sociodemographic and obstetric characteristics based on bivariate test and multivariate logistic regression

Variables/subgroup	Obstructed group (n = 73)	Non obstructed group (n = 73)	Unadjusted p-value	OR (95% CI)	Adjusted p-value	OR (95% CI)
**Age <20 Years**						
Yes	21 (4.2)	5 (13.4)	*<0.001	5.49 (1.94–15.54)	*<0.001	5.43 (1.20–17.13)
No	52 (95.8)	68 (86.6)				
**Booking status**						
Booked	9 (12.3)	70 (95.5)	*<0.001	0.01 (0.00–0.02)	*<0.001	0.01 (0.00–0.02)
Unbooked	64 (87.7)	3 (4.1)				
**Parity**						
Nulliparous	38 (52.1)	15 (20.5)	*<0.001	4.20 (2.02–8.71)	*<0.001	4.15 (2.42–7.25)
Parous	35 (47.9)	58 (79.5)				
Socio-economic class*						
High	19 (28.4)	33 (45.2)	*0.039	0.48 (0.24–0.97)	0.059	0.48 (0.28–0.81)
Low	48 (71.6)	40 (54.8)				
**Maternal height (meters)**						
<1.52	53 (72.6)	4 (5.5)	*<0.001	45.71 (14.24–141.74)	*<0.001	44.74 (19.51–113.53)
≥1.52	20 (27.4)	69 (94.5)				
**Birth weight**						
≤4.0 kg	45 (12.3)	19 (95.5)	*<0.001	4.57 (2.26–9.24)	*<0.001	4.52 (2.69–7.71)
<4.0 kg	28 (87.7)	54 (4.1)				

Conditional logistic regression was employed (p<0.1) in the multivariate analysis to control confounding variables: Teenage age, booking status, parity, socio-economic class, maternal height and birth weight, CI: Confidence interval 95% and OR: Odds ratio

**Table 3:** Causes of obstructed labour observed in the women

Causes	Obstructed group (%)	Non obstructed group (%)
Cephalopelvic disproportion	53 (72.6)	21 (28.8)
Normal	0 (0.0)	45 (61.6)
Occipitoposterior position	9 (12.3)	3 (4.2)
Abnormal lie	6 (8.2)	2 (2.7)
Hydrocephalus	3 (4.1)	0 (0.0)
Uterine fibroids	2 (2.8)	2 (2.7)
Total	73 (100.0)	73 (100.0)

**Table 4:** Type of surgery performed among women with obstructed labour

Type of surgery	Obstructed group (%)	Non obstructed group (%)
Cesarean section	61 (83.6)	30 (41.1)
Spontaneous vaginal delivery	0 (0.0)	41 (56.2)
Laparotomy and repair of uterus	10 (13.7)	0 (0.0)
Laparotomy and subtotal hysterectomy	2 (2.7)	0 (0.0)
Forceps delivery	0 (0.0)	0 (0.0)
Ventouse delivery	0 (0.0)	2 (2.7)
Total	73 (100.0)	73 (100.0)

**Table 5:** Maternal outcome and complications of obstructed labour

Variables	Obstructed group (%)	Non obstructed group (%)
Abdominal distension	15 (20.5)	67 (91.8)
Uterine rupture	11 (15.1)	0 (0.0)
Postpartum haemorrhage	9 (12.3)	2 (2.7)
Puerperal sepsis	4 (5.5)	2 (2.7)
Bladder rupture	2 (2.7)	0 (0.0)
Vesico-vaginal fistula	3 (4.1)	0 (0.0)
Urinary tract infection	5 (6.8)	1 (1.4)
Wound sepsis	7 (9.2)	1 (1.4)
Mortality	3 (4.1)	0 (0.0)

**Table 6:** Fetal outcomes and complications of obstructed labour

Variables	Obstructed group (%)	Non-obstructed group (%)
Normal	17(23.3)	58 (79.5)
Stillbirth	14 (19.2)	3 (4.1)
Neonatal asphyxia	23 (31.5)	4 (5.5)
Neonatal jaundice	10 (13.7)	4(5.5)
Neonatal sepsis	7 (9.6)	3(4.1)
Early neonatal death	2 (2.7)	1(1.3)
Total	73 (100.0)	73 (100.0)

## SIGNIFICANCE STATEMENT

This study discovers the emerging risk factors of obstructed labour to be teenage pregnancy, unbooked status, nulliparity and high birth weight. Complications were observed in 79.5% of the cases of obstructed labour with the emerging complications comprising largely abdominal distension, uterine rupture, postpartum haemorrhage, wound sepsis and puerperal sepsis. Thus a new theory about the obstructed labour may be arrived at and will consider that long labour in low-income countries might be obstructed.
